# The Similarities between Human Mitochondria and Bacteria in the Context of Structure, Genome, and Base Excision Repair System

**DOI:** 10.3390/molecules25122857

**Published:** 2020-06-21

**Authors:** Karolina Boguszewska, Michał Szewczuk, Julia Kaźmierczak-Barańska, Bolesław T. Karwowski

**Affiliations:** DNA Damage Laboratory of Food Science Department, Faculty of Pharmacy, Medical University of Lodz, ul. Muszynskiego 1, 90-151 Lodz, Poland; karolina.boguszewska@stud.umed.lodz.pl (K.B.); michal.szewczuk@stud.umed.lodz.pl (M.S.); julia.kazmierczak-baranska@umed.lodz.pl (J.K.-B.)

**Keywords:** mitochondria, mtDNA, DNA repair, BER, ROS

## Abstract

Mitochondria emerged from bacterial ancestors during endosymbiosis and are crucial for cellular processes such as energy production and homeostasis, stress responses, cell survival, and more. They are the site of aerobic respiration and adenosine triphosphate (ATP) production in eukaryotes. However, oxidative phosphorylation (OXPHOS) is also the source of reactive oxygen species (ROS), which are both important and dangerous for the cell. Human mitochondria contain mitochondrial DNA (mtDNA), and its integrity may be endangered by the action of ROS. Fortunately, human mitochondria have repair mechanisms that allow protecting mtDNA and repairing lesions that may contribute to the occurrence of mutations. Mutagenesis of the mitochondrial genome may manifest in the form of pathological states such as mitochondrial, neurodegenerative, and/or cardiovascular diseases, premature aging, and cancer. The review describes the mitochondrial structure, genome, and the main mitochondrial repair mechanism (base excision repair (BER)) of oxidative lesions in the context of common features between human mitochondria and bacteria. The authors present a holistic view of the similarities of mitochondria and bacteria to show that bacteria may be an interesting experimental model for studying mitochondrial diseases, especially those where the mechanism of DNA repair is impaired.

## 1. Introduction

Mitochondria and mitochondrial DNA (mtDNA) have been of particular interest to researchers in recent years. The endosymbiotic theory of mitochondrial origin is nowadays well confirmed—it took place about 1.5 billion years ago and was related to the increase of O_2_ level in the atmosphere [1]. The theory states that in the general view, mitochondria are ancestors of the ancient endosymbiotic organisms (the host) and the symbiont resembling bacteria as we know them today. The symbiont is believed to be an ancient α-proteobacteria related to *Rickettsiales* lineage that was included as a part of the host [2,3]. The host is believed to be an archaeon, most probably an *Asgard superphylum* archaea [4]. The mitochondria have gone through many evolutionary stages from free-living bacteria to an integrated part of the cell as an organelle. As a result of the endosymbiosis, mitochondria obtained transport proteins, cristae structure, biochemical pathways (e.g., glycolytic pathway and lipid synthesis), and division mechanisms that were integrated between symbiont and host cell [2,5,6]. Moreover, the size of the symbiont’s genome was reduced due to endosymbiotic gene transfer (EGT) or gene loss during evolution. Genes involved in nucleoside and amino acids’ biosynthesis, anaerobic glycolysis, and cellular regulation were conveyed to the host and are currently encoded by nuclear DNA (nDNA) in humans [7]. Mitochondrial symbiosis is considered as the starting point of the eukaryogenesis, as absorption of the symbiont promoted significant evolutionary advantage (e.g., functioning in broader environmental conditions) [8].

Human mitochondria play a crucial role in energy production for the whole organism through the synthesis of adenosine triphosphate (ATP) in the oxidative phosphorylation (OXPHOS). At the same time, a significant amount of reactive oxygen species (ROS), which have a high oxidation potential, is generated. Therefore, mtDNA is particularly vulnerable to oxidative damage. Mutations emerging within mtDNA can impair the energy production and the health of the entire organism, as proteins encoded in the mtDNA are elements of the OXPHOS [9]. Therefore, mitochondria must have efficient repair mechanisms to prevent mutations and permanent changes in their genetic information. Mutations within mtDNA may lead to the onset of mitochondrial diseases, which are a major group of genetic disorders [10,11]. Therefore, a detailed understanding of mitochondrial genetics and repair mechanisms is essential for the development of new therapies. It is assumed that in the mitochondria, the majority of lesions are repaired via the base excision repair (BER) system. It repairs the damage in DNA by cutting out a single nucleobase (short-patch BER, SP-BER) or fragment of 2–10 nucleotides (long-patch BER, LP-BER) [12,13]. As the first step, specific enzymes recognize and remove damaged bases, and then a correct nucleotide is inserted, followed by DNA strand ligation.

This review shows similarities between human mitochondria and bacteria on many levels such as structure, genome, and molecular machinery. The paper includes a description of the human mitochondria, mtDNA, and its oxidative damage, and the BER system operating in this organelle. The BER system is well described throughout the last few decades and is a highly conserved molecular mechanism (somewhat different among species). We bring attention to the bacterial origin of the mitochondrial BER basing on the recent literature and to its impact on the onset of mitochondrial disorders.

## 2. Common Features of Mitochondria and Bacteria

Mitochondria are vital for the majority of eukaryotic organisms and are present in the cytoplasm of almost every cell. They produce energy from nutrients by coupling the pyruvate and fatty acids’ oxidation with the electron transport chain (ETC). Mitochondria are versatile organelle with many crucial functions for the cell—apart from keeping the cell alive by providing the energy; they also regulate redox reactions [14], take part in apoptosis signaling [15], regulate innate immune reactions [16], maintain calcium homeostasis [17], and synthesize Fe/S clusters [18,19]. Here, we focus on the structure and division mechanisms of mitochondria and bacteria to emphasize common features that may be argued in favor of the endosymbiotic theory of mitochondrial origin.

### 2.1. Structure

Mitochondria are usually presented as bacteria-shaped ellipsoids (2–8 μm); however, they may have different shapes and sizes [20]. The number of mitochondria per cell depends on the cell type and the organism type—it can vary in the range of few to a few thousand. Cells that need more energy to operate have a higher number of mitochondria e.g., cardiac muscle or liver cells [21]. The number of mitochondrial proteins also corresponds with cell type and organism—in human cardiac muscles, over 600 proteins were identified [22]. Mitochondria are freely distributed in the cell and create a dynamic network that moves along the microtubule filaments [9,23]. Eukaryotic cells contain three major cytoskeletal systems: microfilaments, microtubules, and intermediate filaments, which are assembled from actin, tubulin, and intermediate filament proteins, respectively. Up to date, those cytoskeletal networks have been associated with different functions of mitochondria such as regulating membrane permeability by tubulins which are located near voltage-dependent actin channels (VDAC) and mitochondrial movement [24].

All human mitochondria have the same basic structures—mitochondrial matrix, intermembrane space, the smooth outer mitochondrial membrane (OMM), and the inner mitochondrial membrane (IMM), which is very convoluted and forms a tubular cristae structure. IMM is where ETC complexes and ATP synthase complex are located. It is also the site of mtDNA repair enzymes action [25]. The OMM consists of a phospholipid bilayer containing integral proteins—porin/VDAC transmembrane canals of about 10 Å diameter allowing nutrients, ions, ATP, and adenosine diphosphate (ADP) to penetrate the membrane [26]. Only oxygen (O_2_), water (H_2_O), carbon dioxide (CO_2_), and hydrogen peroxide (H_2_O_2_) can freely penetrate through the OMM. MtDNA-encoded proteins constitute only a small part of all respiratory enzymes’ subunits; nonetheless, they are essential for the proper operation of the OXPHOS. Other mitochondrial proteins (approximately 1100), of which several hundred are needed for mtDNA expression, are encoded by nDNA [27,28]. Proteins encoded in the nucleus are translated in the cytosol as precursors of mature proteins [29]. They contain targeting signals that are recognized by receptors on the mitochondrial surface. When needed, proteins are recruited and directed to proper mitochondrial destination. The transport of most proteins’ precursors through the OMM into the intermembrane space requires specific protein complexes—translocases of the outer membrane (TOM) [26]. Almost all precursors enter the intermembrane space through TOMs and are subsequently directed to different pathways, which will not be discussed in this review [26]. IMM is impermeable for the majority of molecules. The selective transport of molecules (e.g., ions, ATP, proteins) through IMM requires transporter proteins. This transport machinery is essential for distributing energy within the cell. There are over 30 types of transporters embedded in the IMM [30]. Translocase of the inner membrane (TIM) is one of them [26]. IMM’s impermeability, among others, is caused by the presence of cardiolipin (CL) in its structure. CL is a signature phospholipid for IMM and constitutes almost 20% of its total lipid content [31]. CL seems to be crucial also for overall mitochondria maintenance. It takes part in the organization of channel proteins activity e.g., presequence translocase of the inner membrane (TIM23) complex–presequence translocase-associated motor (PAM) and TOM complexes [26]. Moreover, CL stabilizes OXPHOS complexes, allowing undisturbed ATP synthesis, takes part in fission/fusion dynamics, regulates protein import, and regulates mitophagy [31,32,33]. It is also present in the OMM, in particular at IMM/OMM contact sites [32]. Studies also show that CL impacts membrane folding and cristae formation [34]. The structural complexity of IMM allows the fast distribution of protein complexes in the matrix. Energy production through OXPHOS is the most discussed topic concerning mitochondria. Energy carriers (FAD and NAD^+^) are reduced in biochemical pathways and transport energy to IMM where complexes of OXPHOS are located. Complexes of OXPHOS include approximately 90 proteins encoded by nDNA and mtDNA [35]. The mitochondrial matrix (aqueous space surrounded by IMM) contains enzymes involved in basic metabolic cycles such as β-oxidation and the Krebs cycle (except for the oxidation of succinate to fumarate on the IMM), which provide energy carriers for ETC [35]. Genetic machinery is also located in the matrix and will be discussed further in the text.

Bacterial cells are much smaller than eukaryotic cells, but their size may vary from nano- to millimeters [36]. The standard size of *Escherichia coli* is about 2 μm, while one of the biggest bacterial cells (*Thiomargarita namibiensis*) has a diameter of 0.75 mm. The size of prokaryotic organisms is limited mostly by their nutritional restrictions and the size of molecules diffusing through their membranes [36]. However, there are also theories on bacterial homeostasis, which state that bacteria grow only for a specific time or that they constantly monitor their size and switch off the cell cycle after reaching their critical size [37]. Bacteria have filamentous morphology, which is partially responsible for intracellular transport and cell division, and is associated with resistance to phagocytosis [38]. Communication between individual bacterial cells is crucial for colony survival. The network of chemical signals allows cells to avoid overspreading the colony, which may result in the elevated levels of toxic metabolites and cutting off the supply of nutrients [39,40]. Most bacteria have quite simple cellular structure—in the simplest view, they consist of the cell wall, aqueous cytosol, and genetic material. The majority of bacteria have a cell wall that maintains the shape of bacteria and protects it against osmotic lysis [41]. The rigidity of the cell wall is a result of a peptidoglycan (PG) layer presence. PG is a covalent macromolecular structure of glycan chains (stiff) cross-linked with peptide bridges (flexible) [42]. Two general classes of bacterial cell walls are described. Cell walls of Gram-positive bacteria contain a thick (20–80 nm) PG multilayer with teichoic acids embedded and lipoteichoic acid, which extends to the cytoplasmic membrane. In the case of Gram-negative bacteria, lipoproteins link the outer membrane with a thin (1–7 nm) PG layer located in periplasmic space between the two membranes [42].

Recent years brought to light new facts about lipid species making up bacterial membranes. They tend to coalesce into functional membrane microdomains, which is a phenomenon known as “lipid ordering” [43]. These microdomains contain lipid rafts that have a high density of specific lipids and are a site of bacterial signal transduction cascades and transport proteins. Interestingly, the same lipid structures were observed in eukaryotes long before that, but common opinion at the time was that prokaryotic organisms are not as complex structurally, nor do they need such lipid-rich sites [44]. The lipid content of bacterial lipid rafts is still not fully known. It seems that only a few species (e.g., *Helicobacter pylori*) are able to incorporate cholesterol into its membranes, which is the major lipid for eukaryotic cells. However, bacteria may contain cholesterol-like polyisoprenoid lipids that also assemble microdomains [44]. Lipid rafts consist also of proteins, from which the most important is flotillin. It is considered as a scaffold that favors the recruitment of other proteins. Flotillin keeps lipid rafts functional by promoting the interactions of raft-associated proteins, organizing signaling complexes, and thus, it is essential for cellular processes e.g., signal transduction or membrane sorting [44]. The flotillin is also described in eukaryotic cells, and the lack of it may be related to neurodegenerative diseases [45]. In the case of bacteria, flotillin-like proteins are also present and seem to play the same role as a scaffold for other proteins [44]. The cytosol is the major environment in all bacterial cells. It is an aqueous space that contains a high concentration of ions, metabolites, macromolecules, and molecular assemblies [46]. The cytoplasmic membrane surrounds the cytoplasm and separates it from the extracellular space or the periplasm in Gram-positive or Gram-negative species, respectively. The membrane maintains a proton gradient that drives energy production, the same as in mitochondria. The cytoplasm is not compartmentalized in most bacteria and archaea. Instead, processes occur in specific cytoplasmic regions [47]. In the case of the bacterial cytoskeleton, for many years, it was believed that there is none and that only the cell wall shapes the whole cell [48]. However, homologs of all three eukaryotic cytoskeletal elements have now been identified in bacteria: FtsZ for tubulin, MreB for actin, and crescentin for intermediate filament proteins. FtsZ is the most important one, as it takes part in cell division (discussed in the next section) [42,49]. MreB, a peripheral protein, is a bacterial actin homolog that is widely distributed in rod-shaped bacteria and is involved in cell shape and membrane fluidity maintenance [50]. Moreover, bacteria contain their genetic material in the central region of the cell in the form of the nucleoid, which is presented in detail in Section 3.

Over the years, the increasing number of common features between bacteria and mitochondria has been described [2,51,52,53]. As *Rickettsia*, a commonly accepted mitochondrial ancestor, is described as *coccobacillus*, some authors indicate their anatomical correlation with modern mitochondria, which mostly have a spherical, elongated shape corresponding with the shape of *coccobacillus* [54]. Eukaryotic cytoskeletal elements, which have their bacterial homologs, are detected in mitochondria. Tubulin is present in mitochondrial membranes where it plays a role in mitochondrial permeability mechanics [55]. Interesting, yet not fully explained, is the fact that mitochondria communicate with each other and with other organelles. An example of such communication is the mitochondrial transfer (MT). It may occur between mitochondria (during fusion) or between cells (through nanotube linkages or extracellular vesicles) [56]. Although MT is assumed to be triggered mostly when the cell faces a depletion of functioning mtDNA molecules, the transfer may result also in the spreading of mutations between different tissues [57]. MT aims to restore cellular functions in the case of mtDNA deficiency in the cell and prevent apoptosis or the activation of oncogenes [58,59]. However, examples of MT have been described in mammalian cells concerning carcinogenesis [60]. The transfer occurs especially when tissues of growing tumors lack respiration capacity. Moreover, the transfer of the whole mitochondria from healthy to cancerous cells has been described. It is a mechanism of oxidative stress avoidance that is a major cause of cancer’s resistance to therapies [58,60]. To some extent, this network of signals may be compared to bacterial communication within the colony, which is essential for their survival.

As described previously, mitochondria have two membranes (IMM and OMM) and two aqueous compartments (matrix and intermembrane space), the same as is observed in Gram-positive bacteria such as *Rickettsia* [26]. The fact that mitochondria have two membranes may support the endosymbiotic theory. However, there are also different theories in this matter. It is probable to some extent that one membrane could come from a prokaryotic ancestor and the other could indicate the vesicle that was formed upon the symbiont engulfment [61]. On the other hand, a recent study confirms that protein complexes responsible for cristae formation are of α-proteobacterial origin [62]. To elucidate this issue, more studies must be completed. Mitochondrial membranes (IMM and OMM) have different lipid compositions and asymmetric distribution of lipids, which is considered as a remnant of bacteria symbiosis with the host [63]. CL is recognized as the major lipid in IMM (up to 20% of total lipids); however, it is also present in OMM within contact sites of both membranes where fission/fusion is believed to occur [31]. Bacterial membranes contain lipid rafts with structure and protein composition corresponding to those found in eukaryotic cells. However, the lipid content differs among eukaryotes and bacterial species [32,44]. Eukaryotic membranes contain mainly sterols (e.g., cholesterol) and sphingolipids. In the case of bacteria, only a few Gram-positive bacteria have cholesterol in them, while Gram-negative bacteria contain even three types of cholesterol glycolipids, but they are not able to produce it [43]. The exact lipid content of bacterial membranes is still not fully known, also because it varies among species. Additionally, porins are present in the outer membrane of both bacteria and mitochondria, which allow the transmembrane transport of molecules [42]. Therefore, it seems that mitochondria have some common aspects with bacteria regarding membranes and that structural characteristics such as their double membrane may act in favor of the endosymbiotic theory. Moreover, the molecular machinery within cells, especially ATP synthesis, may also be an argument. OXPHOS operates similarly to bacterial aerobic ATP synthesis; both are located within membranes (IMM in mitochondria and cytoplasmic membrane in bacteria) [64]. However, bacterial ATP synthase has a simpler structure [65]. Furthermore, the majority of proteins present in the mitochondrial matrix (with characteristic Fe/S cluster), which take part in crucial pathways (e.g., Krebs cycle, cofactor biosynthesis, or β-oxidation) have their homologs among bacteria, or their core subunits are of bacterial origin [2,28,66]. In this review, we do not focus on discussing those pathways. Bacteria and mitochondria are also similar in the light of genetic elements—both contain circular DNA and divide through fission, which is presented in the next section.

### 2.2. Fusion/Fission Systems

The mitochondria control system consists of the regulation of mitochondrial fusion/fission dynamics and the regulation of mitochondrial autophagy (mitophagy) [67]. Disruption of those mechanisms may lead to pathological conditions (e.g., metabolic disorder, neurodegeneration) [68,69]. When minor stress affects mitochondrion, it can fuse with another healthy mitochondrion. The fusion process includes sharing undamaged components and in the case of damaged genome fragments, it allows genetic complementation. Fission is important for cellular division and the distribution of mitochondria within the cell, and it occurs also when a mitochondrion gets heavily disrupted; it is subsequently separated and fragmented [69]. Degradation occurs through mitophagy—mitochondria are divided into smaller fragments suitable for autophagosomal encapsulation, and then the undamaged components are recycled for further fusions or the synthesis of new mitochondria. Fusion and fission are regulated transcriptionally and non-transcriptionally by various factors, e.g., cellular stress, redox status, or metabolic and energetic balance within the cell [70]. Since both processes are closely correlated, the balance between them is crucial for the cell functioning [67]. The two pathways are strictly dependent on the presence of lipid rafts along the mitochondrial membrane. These lipid rafts contain e.g., cholesterol and sphingolipids that play a pivotal role in cell signaling [45].

Mitochondrial fusion machinery is controlled by three nuclear guanosine triphosphate proteins (GTPases): mitofusin protein 1 and 2 (MFN1 and MFN2) are located in the OMM and mediate its fusion, while optic atrophy protein 1 (OPA1) is responsible for the fusion of IMM [69]. After two mitochondria get close to each other, the fusion process begins with the recruitment of MFN1 or MFN2 to the lipid rafts localized along the OMM of both mitochondria. Studies show that mitofusin proteins are also essential for OXPHOS and mtDNA maintenance [71]. The lipid fragment of both membranes gets shared and one great mitochondrion is formed, still containing two separated IMMs. Then, OPA1 is recruited and the lipid rafts located along IMMs start to fuse [70]. It is believed that besides the recruitment of necessary proteins, lipid rafts play a crucial role as an optimal environment for chemical reactions underlying the process of membranes fusion. The disruption of lipid microdomains impairs mitochondrial network formation. The function of MFN2 and dynamin-related protein 1 (DRP1) depends on their proper recruitment to lipid rafts, which is essential for the initiation of fusion/fission [67]. During fission, the mitochondrion is divided into daughter mitochondria. The main mediator of the fission process is DRP1, which is recruited from the cytosol to the mitochondrial surface at contact sites with the endoplasmic reticulum. Lipids are responsible for membrane dynamics as they lower the energy barrier for fusion/fission. CL recruits and binds DRP1 and stimulates its GTPase activity. Fission begins with the IMM division inside the mitochondrion and is followed by the OMM division—two daughter mitochondria emerge [23]. This process of division is also crucial for preventing the accumulation of mtDNA mutations, as mtDNA is transferred to the new mitochondrion [69]. Recently, new mechanisms of fission regulation were revealed such as the cooperation of mitochondria and lysosomes or the recruitment of Golgi-related lipid to the fission sites [72,73].

In the case of most bacteria, fusion is not observed. However, bacterial filaments may be treated as equivalent to some extent. Filaments may be a bacterial way to divide or connect with the host [74]. Such filaments are also created upon stress conditions, and it is suspected that filaments may have been present in ancient bacteria species [23]. The situation is different for the fission process, as it occurs in most organisms, including bacteria. The first steps of cytokinesis are DNA replication and nucleoid segregation. Then, a Z-ring is formed—it contains the key division protein FtsZ [75]. The bacterial Z-ring serves as a scaffold to about 20 downstream proteins (e.g., PG synthases and hydrolases), which are responsible for cell wall remodeling [49]. FtsZ (bacterial homolog of tubulin) is conserved in almost all bacteria, many *Archaea*, and the mitochondria of some early-branching eukaryotes [47]. On the other hand, mitochondrial FtsZ is absent from the genomes of yeast, plants, and animals. It is assumed that during the evolution, DRP proteins replaced FtsZ in higher eukaryotes to undertake the process of cell fission [76]. Moreover, a new study confirms that mitochondrial fission machinery is structurally conserved. This hypothesis was tested by creating constructs and directing an α-proteobacterial FtsZ protein to the mitochondrial matrix. The authors show that FtsZ localized at fission sites and increased the rate of mitochondrial fission [77].

## 3. Human Mitochondrial DNA vs. Bacterial DNA

### 3.1. Human Mitochondrial DNA (mtDNA)

Human mtDNA plays an essential role in mitochondrial biogenesis, the maintenance of cell metabolism, and cellular energy homeostasis. Mitochondrial genetic material exists in a form of double-stranded circular molecules of deoxyribonucleic acid. Each human mtDNA molecule consists of 16,569 base pairs and has a molecular mass of 10^7^ Da [78]. The number of mtDNA copies per cell depends on the type of cell in the human body. Energy-consuming cells such as cardiac muscles have 4000–6000 copies of mtDNA per cell, while kidney, liver, or lung cells have less—500–2000 copies per cell. The individual mitochondrion contains normal and mutated mtDNA molecules, the phenomenon which is known as heteroplasmy. Upon fission (e.g., during cell division), daughter mitochondria obtain random copies of mtDNA molecules, including mutated ones [57]. When the number of mutated mtDNA increases, cellular energy production is more likely to be affected. If the ratio of mutated and not mutated mtDNA is too high (threshold value), the odds for the disease occurrence also increase [79]. The mtDNA is packed inside slightly elongated, irregularly shaped protein–DNA complexes called nucleoids. Nucleoids are located in the mitochondrial matrix probably in proximity to IMM. Wang et al. show that some proteins that are known to be embedded in IMM (e.g., adenine nucleotide translocator (ANT) and proteins from complex I of ETC) are identified in the nucleoid, which indicates that nucleoid structures are contiguous to IMM [80]. Moreover, the displacement loop (D-loop) is believed to be anchored in IMM as a central point for nucleoid forming [9,81]. However, it is not yet clear how the nucleoid’s location is regulated. Studies suggest that it may be dependent on the fission and fusion mechanisms [82]. Recent findings also show that a single nucleoid contains a single copy of mtDNA in opposition to previous studies, where up to 8 mtDNA copies were observed in one nucleoid [80,83]. Up to date, around 20 proteins are identified in the mitochondrial nucleoids, among which mitochondrial transcription factor A (TFAM) is the most abundant (approximately 1000 molecules of TFAM per one molecule of mtDNA) [80,82]. It is believed that TFAM is responsible for the compacting of mtDNA, since it causes 90° bends in the DNA and cross-strand binding [82,84]. These compacted structures shield mtDNA and control gene expression. It resembles to some extent the nucleosomes and chromatin structure in the nucleus. Besides mtDNA and TFAM, the nucleoid contains several proteins involved in replication (e.g., mitochondrial DNA polymerase γ (Polγ), replicative DNA helicase (Twinkle), mitochondrial ssDNA-binding protein (mtSSB)), transcription (e.g., DNA-dependent RNA polymerase (POLRMT), mitochondrial transcription factor B2 (TFB2M), mitochondrial transcription elongation factor (TEFM)) and other processes (e.g., RNA helicases, RNA processing proteins, ribosomal proteins, chaperones) [80,82].

Early studies of mtDNA identified the strand-displacement model of replication [85]. Later discoveries of Y arcs and bubble arcs in mtDNA led to the belief that there are other models of replication. Currently, there are several models and their variations proposed, such as the unidirectional RITOLS model (RNA incorporation through the lagging strand) and the bidirectional strand-coupled DNA model [86,87]. It is also reported that in different tissues and/or conditions, different replication models may occur [88]. However, for mitochondria and bacteria comparison, this review focuses only on the strand-displacement model. The replication process starts in the origin sites in both strands and involves a coordinated work of minimum Polγ, Twinkle, mtSSB, topoisomerases, POLRMT, and DNA ligase III (LigIII). Errors occurring during the replication of mtDNA are believed to be the main source of mutations [89]. Polγ is reported to be the only polymerase in mitochondria responsible for replication and mtDNA repair. It is also described as very accurate (less than 10^−6^ errors per nucleotide), which eliminates Polγ as a source of mutations and contradicts previous findings [35]. The mitochondrial gene expression has been recently reviewed by Kotrys et al. Transcription initiation sites are located within the D-loop region of both heavy chain (H, rich in guanine) and light chain (L, rich in cytosine). H chain is a template for the transcription of the majority of mitochondrial genes [90]. Transcription of the L chain results mostly in non-coding RNAs, which are degraded by mitochondrial degradosome (a complex of polynucleotide phosphorylase (PNPT1) and ATP-dependent RNA helicase (SUV3). Two long, polycistronic transcripts are created (from L and H strand promoters: LSP and HSP2, respectively) and subsequently processed to obtain mature RNAs [91,92]. The mitochondrial transcriptional machinery seems to be straightforward [84]. It contains DNA-dependent RNA polymerase (POLRMT) and cofactors: TFAM can unwind mtDNA, initiates the transcription and recruits POLRMT to the site; TFB2M stabilizes the open conformation of the mtDNA strands; TEFM allows strand elongation; mitochondrial transcription termination factor 1 (MTERF1) flips specific bases to terminate transcription [35]. On the other hand, new factors are described as engaged in the transcription e.g., mitochondrial ribosomal protein L7/L12 (MRPL12) or mitochondrial transcription rescue factor 1 (MTRES1), which is also involved in translation through binding to the mitochondrial ribosome. New findings indicate that mitochondrial gene expression processes are yet to be fully understood [90]. Translation in mitochondria consists of standard stages of initiation, elongation, termination, and recycling. The process is crucial for the proper production of enzymes involved in ETC and hence the proper functioning of the organelle. The mammalian mitochondrial ribosome is believed to have bacterial origin, even though some rRNA fragments have been lost during endosymbiosis (e.g., 5S rRNA) [93]. Studies show that missing rRNA fragments are replaced by proteins—the mitochondrial ribosome contains twice as many proteins as cytosolic or bacterial structures [94]. On the other hand, about half of mitochondrial ribosomal proteins do not have bacterial homologs, and it is now clear that the mitoribosome has unique proteins obtained during evolution [93,95]. Details of the mitochondrial translation will not be discussed here, but it is important to remember that disturbances in this process may lead to pathological states [93,96,97].

**Figure 1:**
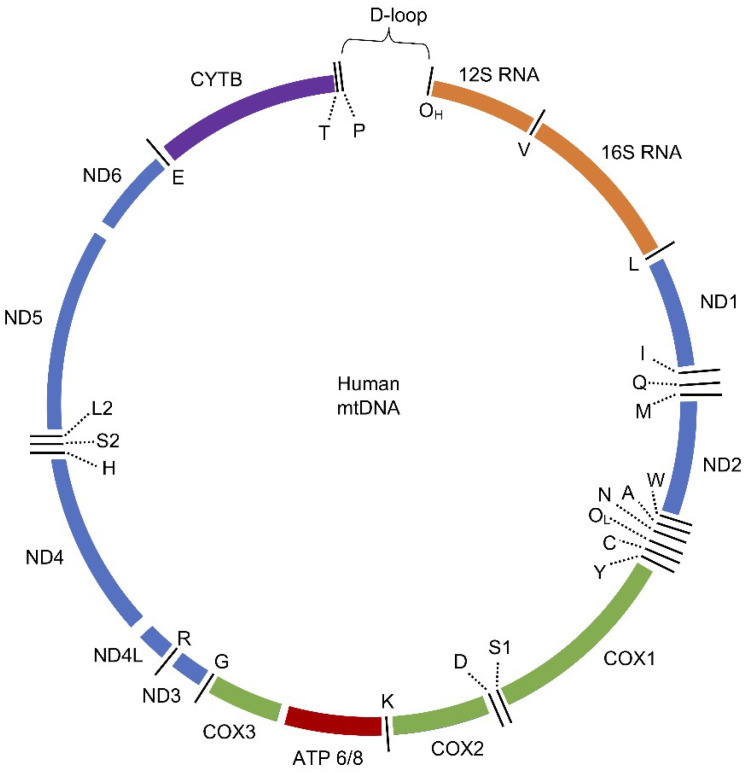
Schematic representation of human mitochondrial DNA (mtDNA). The circular molecule (16,569 bp) contains genes coding for 7 subunits of complex I (ND1–ND6, NADH ubiquinone oxidoreductase chain 1–6) (blue line), 1 subunit of complex III (CYTB, cytochrome B) (purple line), 3 subunits of complex IV (COX1–COX3, cyclooxygenase) (green line), and 2 subunits of complex V (ATP synthase 6 and 8) (red line). Moreover, mtDNA encodes 2 rRNAs (orange line) and 22 tRNAs for specific amino acids (marked with black lines with letter designation for specific amino acids) (based on [9]).

A total of 37 genes are included in mtDNA, encoding 11 mRNA molecules (translated to 13 proteins), 22 tRNA molecules, and 2 rRNA molecules (12S and 16S) (Figure 1). Encoding sequences are embedded in both strands of mtDNA and divide among 12 proteins and 14 tRNAs encoded by the H chain and 1 protein and 8 tRNAs encoded by the L chain [98]. The genetic code of mtDNA is slightly different from nDNA. In nDNA, UGA is a stop codon and in mtDNA, it is coding Trp, AUA is coding Met in mtDNA instead of Ile, and there are four possible stop codons in mtDNA: AGA, AGG, UAA, and UAG [93,99].

Characteristic features of human mtDNA are a lack of introns and high gene density. The exception is the non-coding region (NCR) that contains a D-loop—the starting point of replication and promoters for transcription. D-loop contains hypervariable fragments that differ among individuals, which is useful for population genetics and forensic medicine [100]. All 13 mitochondrial proteins take part in ATP generation by OXPHOS. These proteins are membrane subunits of the respiratory chain: 7 of 45 subunits of complex I (NADH: ubiquinone oxidoreductase), 1 of the 11 subunits of complex III (ubiquinol:cytochrome C oxidoreductase), 3 of 14 subunits of complex IV (cytochrome C oxidase), and 2 of 16 subunits of complex V (ATP synthase) [101,102]. Details of why the OXPHOS subunits in humans are encoded by mtDNA are yet unclear [16,35,78,98]. Two hypotheses give reasons for this phenomenon. One of the hypothesis is that to efficiently regulate ETC, some genes must be expressed “on site”. In the case of changes in the redox state, the adjustment of OXPHOS complexes is needed. Therefore, when mtDNA is present “on site”, mitochondria may rapidly regulate protein expression to prevent malfunctions of ETC, excessive ROS production, and subsequent damage to the cell [103]. The second hypothesis, which was recently revisited, stresses the fact that the majority of mtDNA-encoded proteins are large and hydrophobic. Hence, if expressed in the nucleus, they would be most probably directed to the endoplasmic reticulum instead of mitochondria [104]. It is worth mentioning that both theories are not exclusive, but further studies in this field are necessary to fully understand the reasons why mitochondria need its genetic material [2].

### 3.2. Comparison of Human mtDNA and Bacterial DNA

Since the endosymbiotic theory describes α-proteobacteria as an ancestor of mitochondria, many common features have been discovered over the years between mitochondrial and prokaryotic genomes. The bacterial genomes size varies in the range of approximately 1000–9000 kbp [105]. The genome of *Escherichia coli* is described as 4700 kbp, double-stranded, circular DNA (circular bacterial chromosome), and it is the most popular model for molecular studies [106]. During the endosymbiotic process, a significant reduction of bacterial genome size occurred as a result of gene transfer to the host. Hence, mtDNA has a much smaller size than the average bacterial cell, and it lost many functional genes [107]. The bacterial genome is located in the central part of bacteria in the form of compacted nucleoid. In some bacteria, such as *E. coli*, DNA is supported by multiple plasmids located nearby that encode e.g., antibiotic resistance genes. Human mitochondria have many copies of double-stranded, circular DNA [39]. Same as in bacteria, mtDNA is associated with proteins and shaped into nucleoids, which indicates a possible evolutionary correlation of the two [9]. In the case of mitochondria, TFAM proteins are the main components that compact DNA. The same phenomenon is observed in bacteria where the set of nucleoid-associated proteins (NAPs) compacts genofor into nucleoid [108,109]. They are able to bind to DNA and change its structure. Two major DNA binding proteins involved in this process are DNA-binding protein HU and histone-like nucleoid-structuring (H-NS) protein. They have different functions within the bacterial genome: HU takes part in transcription and cellular growth, while H-NS silences transcription and may arrest the cell cycle [108]. Both proteins are involved in the compacting of the bacterial DNA, and by regulating the dynamics of the nucleoid, they influence gene expression [47]. Bacterial DNA contains mostly functional regions that encode proteins [105]. Therefore, usually, its genome size corresponds with proteome size [110]. The length of DNA in bacteria is also associated with its content of G:::C pairs, which is beneficial for the cell as it decreases the chance of denaturing [111]. Characteristic for bacterial DNA are also conserved unmethylated CpG motifs. They are also described within mtDNA, which is one more evidence for the evolutionary connection of bacterial and mitochondrial genomes [16].

Gene expression machinery in mitochondria is distinct from nuclear expression, which also aligns with the endosymbiotic theory of mitochondrial origin. On the other hand, many of the proteins involved in genome maintenance correspond not with bacterial but with bacteriophage lineage (e.g., POLMRT, Twinkle) [35]. POLRMT is homologous to T7-like bacteriophage’s polymerase. It may indicate that the polymerase-based transcription system was incorporated in eukaryotes early in the evolutionary process or that the proteobacterial system was replaced by phages in the process of evolution [84]. On the other hand, Polγ belongs to the family-A DNA polymerases to which the bacterial DNA polymerase I (PolI) also belongs [35]. The bacterial chromosome is replicated bidirectionally starting from the site of origin and transcription and translation occur simultaneously during the cell cycle. These processes are well known and will not be described here in detail [112,113,114,115]. The replication of prokaryotic and mitochondrial DNA has a different course. The bacterial replication is bidirectional and starts at the same time on both strands. The replication of mtDNA is more complex and requires partial replication of the H strand to activate the process on the L strand [35,116]. Mitochondria contain their own transcriptional and translational machinery—genes are transcribed as mRNA, which is subjected to polyadenylation as post-transcriptional processing [95]. It is another indicator that the mitochondria, as a semi-autonomous organelle, may be a remnant of bacteria in the cell. Until recently, it was believed that mitochondrial transcription machinery is distinct from the whole cell and operates on its own. What supported this theory was that mitochondria create polycistronic transcripts, as bacteria do and have clustered RNA genes, which are typical for eubacteria [7]. In addition, TFB2M is homologous to the rRNA methyltransferase family in bacteria and archaea [35]. However, a new study shows that nuclear regulatory factors also take part in the regulation of mitochondrial gene expression [117].

Taking into consideration common structural and genetic features linking mitochondria and bacteria, it may be assumed that the endosymbiotic theory is correct in the light of current knowledge. The mitochondrial genome seems to be a direct remnant of its prokaryotic ancestor despite differences that arose throughout evolution. Therefore, it may be suspected that other molecular machinery such as DNA repair in mitochondria may also have a bacterial origin. However, the presence of the genetic material in mitochondria has its evolutionary consequences. The major drawback is that mtDNA is exposed to ROS action and hence is highly vulnerable to oxidative DNA damage formation. The next section describes oxidative DNA damage, its formation within mitochondria, and mechanisms of mitochondrial redox homeostasis regulation.

## 4. DNA Damage in Human Mitochondria

### 4.1. Oxidative DNA Damage

The mitochondrial genome is subjected to the same damaging factors as nDNA and even more. DNA damage may occur as single nucleotide damage (e.g., 8-oxoguanine (8-oxo-G)), structural damage (e.g., intrastrand/interstrand cross-linking) or damage to DNA core (e.g., apurinic/apyrimidinic (AP) site). The most frequently mentioned DNA damage types are the oxidation of bases, single-strand breaks (SSBs, e.g., as a result of ROS), the alkylation or deamination of bases, insertion of the incorrect base during replication, the formation of pyrimidine dimers (e.g., as a result of UV radiation), double-strand breaks (DSBs, e.g., as a result of ionizing radiation), the formation of base derivatives (e.g., as a result of H_2_O_2_ or polycyclic hydrocarbons present in smoke), the formation of cross-links between DNA strands (e.g., as a result of vinyl chloride) [118]. DNA damage is also divided into endogenous or exogenous. Endogenous damage is caused by the products of cellular metabolic processes (e.g., ROS and reactive nitrogen species (RNS)) or by the errors emerging during replication. Exogenous damage is caused by external factors e.g., UV radiation, high temperature, toxins of plant origin, aromatic mutagens, or radio- and chemotherapy. Ionizing radiation may lead to the formation of cracks in both DNA strands and complex lesions that are a characteristic feature for radiation. As a result of UV light, pyrimidine dimers may be formed between adjacent cytosine (C) and thymine (T) or two T residues [14]. The likelihood of the oxidative damage in mtDNA is the highest of all cellular locations due to OXPHOS reactions which occur close to mitochondrial genetic material. Furthermore, in the process of mtDNA replication, the H-chain is separated for some time, making it more vulnerable to mutagenic agents [78].

To date, more than 100 different types of oxidative DNA damage have been identified [119]. Each nucleobase is vulnerable to oxidative damage. Changes in base structures lead to transversion or transitions in mtDNA, which causes mutations and subsequent diseases. The most frequently oxidized base is guanine (G) due to its low oxidation potential: it occurs 10^5^ times per day/per cell [120]. The 8-oxo-G is generated through the oxidation of C8 in G and its presence leads to G:::C—> A::T transversions. 8-oxo-G may be further oxidized to the form of 2,6-diamino-4-hydroxy-5-formamidopyrimidine (FapyG), which is reported to be even more mutagenic [121]. 8-oxo-G is well described and is often used in studies on repair systems in cells due to its chemical stability. Adenine (A) undergoes similar conversions; however, the yield of 8-oxo-A and 4,6-diamino-5-formamidopyrimidine (FapyA) formation is lower than for G. 8-oxo-A and FapyA lead to A::T—> G:::C transversions. FapyA is one of the most frequent A lesions resulting from radiation. The mutagenic potential of A-derived damage is lower than those of G due to its lower structural stability in the DNA strand. Thymine glycol (TG) is the most frequent lesion of T, which blocks polymerase activity during replication [122]. Other examples of T lesions are 5,6-dihydrothymine (DTH) and 5-hydroxymethyluracil (5HmU). While the first one is not reported to be cytotoxic or mutagenic, the latter causes T::A—>G:::C transitions. Oxidative products of C are 5-hydroxy-2′-deoxycytidine (OH5C), which leads to C:::G—>T::A transitions and 5-methylcytosine (5mC) which, apart from lesion forming potential, has a gene regulation function [119]. Oxidative DNA lesions are mostly formed as a result of ROS action. Taking into consideration that proteins involved in OXPHOS are encoded by mtDNA and are on the “first line” of exposure, the proper redox homeostasis is crucial for the organism to support mitochondrial genome integrity [123].

### 4.2. Mitochondrial ROS Production and Redox Homeostasis

MtDNA is highly sensitive to oxidative lesions; therefore, the mutagenesis rate in mtDNA may be even 10 to 20-fold higher than in nDNA [78,124]. The mtDNA seems to be an easy target for the free radicals due to a lack of chromatin structure (protection of histones). However, TFAM must be mentioned here, as the protein is able to compact mtDNA into a nucleoid [35]. Nucleoids resemble the tight structure of chromatin; thus, it is assumed that mtDNA is shielded from the action of ROS at least until the moment of loosening the structure before replication or transcription. TFAM also operates in coordination with POLRMT during transcription to bypass oxidative damage (e.g., 8-oxo-G), which otherwise would stop the process [125]. Apart from the mutagenic potential, ROS are important regulators of the molecular pathways (e.g., gene expression or cell differentiation) [126]. ROS are mainly generated during physiological processes, the metabolism of chemical compounds, or as a response to a stress factor (e.g., ionizing radiation). Endogenously, radicals are formed through enzymatic cellular systems such as cytochrome P450 reductase, nitric oxide synthase (NOS), or as a result of “electron leakage” in ETC [127,128]. Respiration generates a high amount of ROS (about 90%) [129]. They include H_2_O_2_, superoxide anion (O_2_^•−^), singlet oxygen (^1^O_2_), ozone (O_3_), and hydroxyl radical (^•^OH). ROS are produced mostly as a result of the reaction between O_2_ and electrons, which come mostly from complexes I and III of ETC. Some electrons “leak” from the ETC before full, four-electron reduction, causing a direct reduction of O_2_ to O_2_^•−^ that is the most devastating radical generated in high quantities (2–3 nmol/min/mg of protein) [130]. IMM is impermeable for O_2_^•−^. The anion formed by complex I is directed into the mitochondrial matrix, but complex III may direct some amount also into the intermembrane space [128].

**Figure 2:**
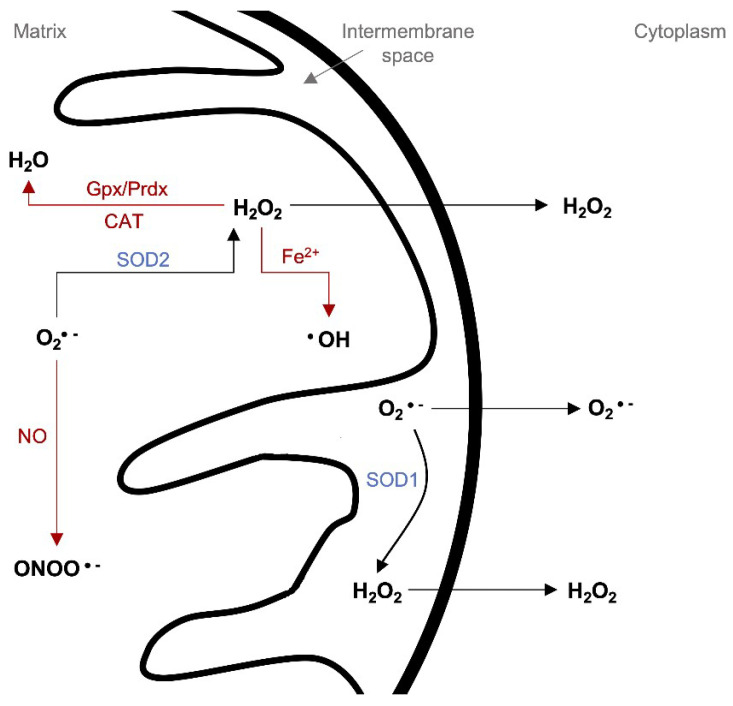
Schematic representation of reactive oxygen species (ROS) signaling in human mitochondria. Superoxide anion (O_2_^•−^) emerges in the mitochondrial matrix and intermembrane space and can be converted to hydrogen peroxide (H_2_O_2_) by superoxide dismutase (SOD1 or SOD2). H_2_O_2_ gets through the membrane into the cytoplasm. O_2_^•−^ may also react with nitric oxide (NO) forming peroxynitrite (ONOO^•−^), which blocks O_2_^•−^ conversion into H_2_O_2_. In the presence of metal ions (Fe^2+^), H_2_O_2_ generates hydroxyl radical (^•^OH). Hydrogen peroxide may be transformed into the water by enzymes: catalase (CAT), glutathione peroxidase (Gpx), or peroxiredoxins (Prdx) (based on [144]).

Free radicals are neutralized in mitochondria by a variety of endogenous enzymatic (e.g., superoxide dismutase (SOD), catalase (CAT), glutathione peroxidase (Gpx), peroxiredoxin (Prdx), hemeoxygenase-1 (HO-1), and thioredoxin (Trx)) and non-enzymatic (e.g., urate, ascorbate, glutathione (GSH), β-carotene, and flavonoids/polyphenols) antioxidants [131,132]. SOD seems to be the first line of defense against ROS (Figure 2). It neutralizes O_2_^•−^ through its degradation to O_2_ and H_2_O_2_. The *SOD1* gene is located on chromosome 21q22.1. SOD1 overexpression may lead to an increase in the ROS level in the cell. Available results indicate that in patients with chromosome 21 trisomy (Down Syndrome, DS), SOD1 levels are elevated in different types of cells and organs. In addition to morphological disorders, DS is associated with impaired learning, memory, and mental development. Genes actively involved in the degeneration of nerve tissue, among others, have been identified as responsible for cognitive functions such as learning, memory, or behavioral disorders. The most commonly described genes in this context include *Sod1*, *App*, or *Sim2* [133]. Moreover, an important factor associated with DS pathologies is oxidative stress [134]. Trisomy 21 is associated with the impairment of mitochondrial function, which leads to an increased level of free radicals and oxidative stress. However, there is no clear evidence of whether SOD1 overexpression is directly responsible or it is rather the result of damaged mitochondria in the DS patient’s brain. There are two types of SOD in mitochondria—SOD1 in the intermembrane space and SOD2 in the mitochondrial matrix [129]. Gulesserian et al. observed differences in SOD expression in the brain of DS patients compared to Alzheimer patients. SOD1 levels in DS were significantly elevated, while in the case of Alzheimer’s disease, they were lowered. Interestingly, SOD2, which is necessary for the proper functioning of mitochondria and neurite growth, did not show significant differences in its amount or distribution in the brain [135]. The authors conclude that SOD1 overexpression in DS may be neuroprotective and constitute a compensatory mechanism for increased oxidative stress, which is also supported by other authors [136,137]. However, different studies show that SOD1 overexpression may partially cause the DS [138].

H_2_O_2_ has moderate reactivity and is not a direct threat to macromolecules, but in metal-catalyzed reactions, it is converted to ^•^OH. The hydroxyl radical is the most reactive radical that causes nucleobases modifications impacting DNA structure [139]. Therefore, H_2_O_2_ may be converted to O_2_ and H_2_O by CAT or Gpx/GSH [129]. CAT is less efficient than GSH, but while GSH is a major antioxidant for the entire cell, in mitochondria, it occurs in low quantities. Gpx utilizes GSH to enable the transformation of H_2_O_2_ to H_2_O; then, GSH is regenerated by glutathione reductase (GR). The Prdx/Trx system operates similarly—Prdx reduces H_2_O_2_ to O_2_ and utilizes Trx to regenerate itself [129]. The oxygen level is controlled by specific hypoxia-inducible transcription factors (HIFs). They inhibit ROS generation by the regulation of acetyl–CoA synthesis, mitochondrial proteins synthesis, and mitophagy. A decreased level of O_2_ leads to HIFs activation, which subsequently leads to a decrease in the production of mtROS or an increase in the production of ROS scavengers. HIFs occur in abundance in tumor tissues that are rich in hypoxic areas. Therefore, HIFs inhibition may serve as a potential therapeutic target in the inhibition of tumor growth [140]. The consequences of ROS in cells are dependent on their concentration. In non-stress conditions, when the ROS level is well-regulated, the redox signaling maintains physiological cell functions, such as cellular differentiation and tissue regeneration. However, in the case of ROS overgeneration or the inhibition of ROS scavengers, the intracellular redox homeostasis is disrupted. It leads to the state of oxidative stress, which is the combination of the impaired ability of tissues to detoxify/compensate overall damage with an overgeneration of ROS. Oxidative stress may induce iron ions, which in turn catalyze the formation of highly reactive ^•^OH [131]. The accumulation of ROS can also disrupt the mitochondrial membrane stability and lead to the activation of the fusion/fission control system or mitophagy [69,141]. Oxidative stress is the main source of SSBs and DSBs, which are the most abundant damage in mtDNA. They impair mitochondria functions through decreasing the ETC protein activity [98]. ROS action may result in substitutions, deletions, and missense mutations at a lower concentration in mtDNA than in nDNA [78]. Experiments on rat cardiomyocytes show that after treating cells with H_2_O_2_, the oxidative stress arises and causes a decrease in the activity of complexes I, III, and IV by 50% within 10 min [9].

Furthermore, oxidative stress induces lipid peroxidation. As a result, reactive lipid peroxidation products are generated e.g., 4-hydroxy-2-nonenal (4-HNE). 4-HNE is produced mainly in mitochondria and is considered as an oxidative stress biomarker. It may form adducts with DNA, proteins, and membrane phospholipids, especially within mitochondria [142]. Studies show that such adducts lead to the onset of cancer, mtDNA damage, reduced membrane integrity, reduced ETC activity, and apoptosis (e.g., through the regulation of proteins involved in cellular stress response to UV, H_2_O_2_, or oxidants) [142,143]. 4-HNE may form HNE-DNA bulky adducts and/or bond with all nucleobases (it reacts with the highest efficiency with G and C). The adduct of 4-HNE and G may be removed from DNA by BER, nucleotide excision repair (NER), or recombination [142]. In the case of nDNA, 4-HNE-dG plays a crucial role in human cancers as it causes p53 mutation and subsequently may lead to the death of cancerous cells [142]. The exact role of 4-HNE-dG within mtDNA is not yet described, but the interaction of 4-HNE with mitochondrial macromolecules is considered as a potential target for cancer treatment.

It is worth mentioning that similar processes of the generation and neutralization of free radicals occur in aerobic bacteria, where ROS also play an important role in their metabolism and emerges as a result of stressors (e.g., exposure to antibiotics) [145]. A model organism for such discussion is *E. coli* (facultative anaerobe) [146]. A four-electron reduction of O_2_ is observed in bacteria, resulting in ROS production. *E. coli* cell contains a set of scavenging enzymes: three types of SOD (Fe-SOD, Mn-SOD, and CuZn-SOD), 2 types of CAT, and alkyl hydroperoxide reductase (Ahp, which reduces H_2_O_2_ to H_2_O). Those enzymes regulate intracellular ROS concentrations, but in the case of additional, exogenous stress factors and higher ROS levels, they are insufficient. Therefore, bacteria have two systems neutralizing exogenous oxidative stress (OxyR and SoxRS), which will not be discussed here [146]. ROS cause serious consequences for bacteria. They impair amino acids, lipids, DNA, or proteins, which may lead to the accumulation of ROS and subsequent cell death. As previously mentioned, 4-HNE is a result of ROS reactions with mitochondrial lipids. It is mutagenic in both mammalian and bacterial cells [142]. During oxidative stress, levels of 4-HNE may increase up to 50 μM and form adducts with DNA [147]. As a response to such DNA damage, the SOS system may be triggered, which is a main system activated in the case of UV action upon DNA. Bacterial defense mechanism concerns also repair of damaged DNA, which is discussed further in this review. In *E. coli*, 4-HNE adducts may be repaired by NER or homologous recombination [147]. The interesting fact is that in the light of ROS, bacteria and mitochondria suffer comparable damage and have similar mechanisms of protection.

The impact of mitochondrial ROS (mtROS) on the cell and human health is considered to be complex. Oxidative stress may cause cell death due to interactions of ROS with cellular proteins, lipids, and DNA [16]. Moreover, mtROS may target DNA repair proteins, which impairs repair systems and increases the number of DNA lesions in the mitochondrial genome [148]. The main repair system in mitochondria is BER, which targets oxidative lesions in mtDNA. Therefore, it is essential that the repair mechanisms within the cell operate properly. A better understanding of the BER mechanism and its elements will allow preventing genomic instability and later diseases.

## 5. Mitochondrial BER System

Every living cell is constantly exposed to many factors, both internal and external, that interact with its genetic material. Products of cellular metabolism, ROS, environmental factors, food contaminants, ionizing radiation, and chemotherapeutics can cause DNA damage (2–7·10^4^ lesions forming per day/per human cell) [148]. The lesions can block gene transcription and lead to mutations. Therefore, cellular repair systems must be constantly active, detect, and mitigate the effects of damage. The effectiveness of repair mechanisms depends on many factors, including the type of cell and the type of damage. In a situation where there are too many lesions or DNA repair systems that do not function effectively, the cell enters the path of apoptosis. Repair systems observed in mitochondria are base excision repair (BER), mismatch repair (MMR), and non-homologous end-joining (NHEJ) [149]. Homologous recombination (HR) is essential for mtDNA repair in plants and yeast, but it is not confirmed to operate in human mitochondria. In addition, nucleotide excision repair (NER) action is not identified in mitochondria [124]. BER is recognized as a predominant system in mitochondria and repairs lesions resulting from oxidation, deamination, alkylation, and SSBs [78]. It is worth noting that the repair process of mtDNA is slower than nDNA, and any defects in its operation may cause serious consequences for the entire cell. On the other hand, errors may also occur as a result of incorrect polymerase action during BER. The polymerase may mispair a base opposite the original lesion site, which often goes undetected and results in mutations. A majority of these mutations are single nucleotide deletions—it is the most common error in BER system [150]. To avoid genetic instability in mitochondria and an impaired action of respiratory pathways, the cell must be ready to repair occurring damage in a highly controlled way.

### 5.1. Mitochondrial BER Overview

**Figure 3:**
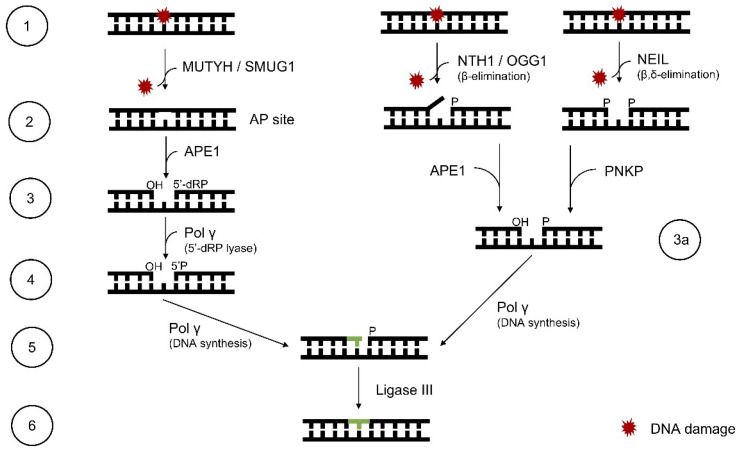
The base excision repair (BER) system in mitochondria. (1) The first step is the action of DNA glycosylases. Depending on the type of damage, different glycosylases are recruited: monofunctional—molecule and adenine DNA glycosylase (MUTYH), single-strand selective monofunctional uracil DNA glycosylase (SMUG1), or bifunctional—endonuclease III homolog 1 (NTH1), 8-oxo-G DNA glycosylase 1 (OGG1), Nei-like 1 (NEIL). DNA glycosylase cleaves the β-N-glycosidic bond and releases the damaged base. (2) In the case of monofunctional glycosylases, an apurinic/apyrimidinic (AP) site is created. Bifunctional glycosylases have AP endonuclease activity—they create a gap in the strand without AP endonuclease. (3) The AP site is incised by AP endonuclease (APE1). (3a) The AP site is processed by APE1 or polynucleotide kinase 3’-phosphatase (PNKP). (4) DNA polymerase γ (Polγ) processes the 5′-end of the strand. (5) The gap in the strand is filled with correct nucleotide by Polγ. (6) Strand is ligated by DNA ligase III (based on [78]).

The BER system is designed to repair damaged DNA by cutting out a single base (SP-BER) or a fragment of 2–10 nucleotides (LP-BER). The ability to excise a single base is a feature that distinguishes BER from other DNA repair mechanisms [151]. The BER system removes about 10^4^ lesions per day in human cells and is based on the few steps (Figure 3): recognition of the lesion by specific glycosylases, excision of the damaged base, AP site removal, processing of strand ends, insertion of the correct nucleotide, and strands ligation [152].

Most of the lesions affect the spatial arrangement of the DNA helix, which improves the detection of the lesion by the cell. Specific repair proteins are recruited to bind to the damaged site so that subsequent repair elements can operate. Enzyme complexes consist of different proteins depending on the type of cell, type of damage, and the phase of the cell cycle. DNA glycosylases find the damaged site and cleave the β-N-glycosidic bond between the base and deoxyribose. This creates an AP site that is recognized by the AP endonuclease [153]. Various glycosylases are known, each of which is responsible for identifying a different type of damage. There are monofunctional glycosylases that cross β-N-glycosidic bonds and bifunctional glycosylases, which additionally can remove AP sites (by β-elimination or β,γ-elimination), resulting in a gap of only one nucleotide (they create SSB). Examples of mitochondrial monofunctional enzymes are uracil DNA glycosylase (UDG), which finds and excises uracil (U) from a single- or double-stranded DNA (ssDNA or dsDNA) molecule and adenine DNA glycosylase (MUTYH), which removes A mispaired with oxidized G. The AP endonuclease (APE1) hydrolyzes the phosphodiester linkage 5′ from the AP site and thereby cleaves the DNA strand. Bifunctional glycosylases such as endonuclease III homolog 1 (NTH1) carries out both stages—recognition and removal of the lesion and AP site cleavage. However, to properly process strand ends, APE1 and polynucleotide kinase 3′-phosphatase (PNKP) are also needed. The free 3′-OH end formed this way allows DNA polymerase γ (Polγ) to add the missing nucleotide. Polγ is still commonly accepted as the only one (from 17) mammalian polymerase reported functioning in mitochondria, despite recent advances in the field [90,154]. BER in mitochondria resembles the one in the nucleus. For SP-BER, a single nucleotide is inserted, while for LP-BER, a longer fragment is inserted: the flap structure. Before the strands are ligated, the flap fragment is cut off by flap structure-specific endonuclease 1 (FEN1) with the assistance of exo/endonuclease G (EXOG1) [155]. The last few years revealed an alternative LP-BER pathway. It does not involve FEN1 action and instead, it employs DNA replication helicase/nuclease 2 (DNA2). DNA2 can stimulate Polγ on its own or in coordination with FEN1 [149,156]. Eventually, a fragment of the DNA chain is “replaced” with a new one. The final stage of the BER mechanism is the joining of the new nucleotide fragment with the “old” strand by the action of LigIII [155]. At first, it was believed that in mitochondria, only SP-BER is present, but studies have already confirmed the activity of LP-BER. The removal of 2-deoxyribonolactone (dL) may be an example. DL covalently binds to Polγ and blocks the SP-BER pathway. However, experiments with dL show that it is removed from mtDNA with the FEN1 endonuclease, which is the key protein in the LP-BER pathway [149]. LP-BER allows repairing more complex lesions such as tandem (lesions of two subsequent nucleobases) and/or clustered lesions (2 or more lesions present within 1–2 turns of the helix) [157]. However, the presence of accumulated damage sites may reduce the activity of BER enzymes [158]. On the other hand, it is reported that in the case of clustered lesions, they are preferentially repaired one by one, mainly by SP-BER, to avoid possible errors or DSBs while cutting out the nucleotide fragments on both strands [159]. Besides, depending on the type and mutual location of damage, only the first lesion can be effectively repaired. TFAM is also believed to be connected to BER action [160]. As BER acts on mtDNA, which is located close to the IMM and compacted within the nucleoid, the theory seems to be probable. Studies show that TFAM binds also to DNA containing 8-oxo-G and that it inhibits BER action in mtDNA. It is suggested that this inhibition is a necessary step for the mitochondria to “assess” the extent of damage and have time to import repair proteins from cytosol [155]. Mitochondrial glycosylases are encoded by nDNA, and those containing the mitochondrial targeting signal (MTS) are translocated into mitochondria [161,162]. The evidence is still limited, but some studies show that repair proteins are imported to mitochondria only in response to stress signals [155]. It is believed that the BER system has a bacterial origin, as the organelle itself [124]. Hence, the next chapter describes human mitochondrial and bacterial BER proteins and stresses their homology and overlapping functions.

### 5.2. BER Proteins in Human Mitochondria and Bacteria

The BER system is highly conserved throughout all organisms [124]. Bacterial DNA repair systems differ according to the species of bacteria and are dependent on the environment they inhabit [163]. However, the bacterial BER corresponds with the mitochondrial one. This repair pathway is crucial for maintaining genetic stability, especially in the context of oxidative damage. Enzymes responsible for repairing DNA in mitochondria and bacteria show common characteristics and functions. Moreover, bacteria are evolutionarily older than mitochondria, and the majority of bacterial BER proteins have homologs in human mitochondria (Table 1). Therefore, it should be considered as at least partial evidence for their shared origin, which supports to some extent the evolutionary theory of the bacterial origin of mitochondria.

The most important proteins in the BER pathway are glycosylases, as they initiate the repair process through detecting and the subsequent excision of DNA lesions. Glycosylases are divided into 4 families: the UDG family (contains characteristic α/β fold), HtH family (contains characteristic helix-hairpin-helix structure), 3-methyl-purine (MPG) family also known as alkyladenine DNA (AAG) family (focused on alkylation damage), and formamidopyrimidine-DNA glycosylase (Fpg)/endonuclease VIII (Nei) family (contains characteristic helix-two turn-helix structure) [162]. Human mitochondria contain a set of glycosylases—UNG1, MUTYH, N-methylpurine DNA glycosylase (AAG/MPG), 8-oxoguanine glycosylase (OGG1), NTH1, and Nei-like 1 and 2 (NEIL1 and NEIL2) [78,124]. Bacteria also contain a set of glycosylases: Ung, Mug, endonucleases III (Nth), alkyladenine DNA glycosylases (AlkA), adenine DNA glycosylase (MutY), 8-oxo-dGTP diphosphatase (MutT), MutM (also known as Fpg), Nei, and N-methylpurine-DNA glycosylase II (MpgII) [151,152].

The first glycosylase family (UDG family) contains 6 subfamilies, of which only the uracil N-glycosylase (UNG) subfamily is found in mitochondria [161]. Its mitochondrial isoform (UNG1) has a 30 amino acid MTS sequence, which is cleaved after entering IMM. UNG1 and its bacterial homolog Ung are monofunctional conserved enzymes that have the same functionality—the excision of U from ss- and dsDNA [161]. U, in this case, may be paired with any canonical base. Interestingly, Ung was the first glycosylase ever discovered in *E. coli* by Lindahl [164]. Human *UNG* and *E. coli Ung* genes show over 55% identity and are believed to be one of the most ancient glycosylase genes [165].

The HtH family of glycosylases includes six subfamilies: Nth, MutY, AlkA, MpgII, 8-oxo-G DNA glycosylase 1 (Ogg1), 8-oxo-G DNA glycosylase 2 (Ogg2), and OGG1. As the most abundant damage excised by the BER system is 8-oxo-G, its repair is studied extensively. The first described repair system that removed 8-oxo-G from DNA was recognized in *E. coli* and named the “GO-system”. It involved 3 repair enzymes: MutT (captures free, damaged bases from nucleotide pool to prevent their incorporation to the DNA strand), MutM (cleaves 8-oxo-G from DNA), and MutY (removes A mispaired with 8-oxo-G) [119]. In human mitochondria, the repair of 8-oxo-G is initiated by OGG1 [161]. OGG1 is a bifunctional enzyme with N-terminal MTS. It has isoforms specific for mitochondrial repair (OGG1-1b and c, OGG1-2a-2e), but their individual roles are still not fully understood [155,161]. Experiments on mitochondrial extracts of *ogg1^(−/−)^* mice show a higher amount of 8-oxo-G in mitochondria compared to the nucleus. It implies that nDNA has alternative repair pathways for such damage that are not present in mitochondria [166]. OGG1 also repairs a product of 8-oxo-G oxidation—FapyG. Glycosylases repairing 8-oxo-G in mitochondria have their bacterial homologs; for example, MutY corresponds with MUTYH [167]. Interestingly, MutY, the representative of MutY subfamily, is the first described enzyme that cleaves undamaged nucleobases—A mismatched with G. Monofunctional MUTYH has the same role of removing mismatched A from A::8-oxo-G, A::G, or A::C pairs. Among about 15 transcripts of the MUTYH enzyme, only one is present in mitochondria—MUTYH-α3 with 14 amino acid MTS [161]. Another pair of homologs is 7,8-dihydro-8-guanine triphosphatase (MTH1, also known as NUDT1) and bacterial MutT. MTH1 neutralizes oxidized bases (e.g., 8-oxo-dGTP) from a nucleotide pool, preventing their incorporation into DNA during replication [168]. However, MTH1 was also found in mitochondria as an additional enzyme excising 8-oxo-G in the case of high 8-oxo-G concentration [169]. NTH1, a bifunctional glycosylase, is also expressed in the mitochondria and named after its bacterial prototype Nth [161]. It recognizes lesions such as TG, 5-hydroxycytosine (5hC), 5-hydroxyuracil (5hU), and Fapy.

Monofunctional glycosylases from the AAG family excise deaminated and/or alkylated bases—3mA, 1-methylguanine (1mG), 7-methylguanine (7mG), hypoxanthine, and etheno adducts in ss- and ds-DNA. Two isoforms are identified in mitochondria: AAG-A (17 amino acid MTS) and AAG-B (12 amino acid MTS) [161]. AlkA glycosylase is present in many bacteria species and corresponds with the AAG mitochondrial enzymes. Bacterial AlkA lacks one HtH motif in its structure, but it recognizes the same substrates as AAG [161]. *E. coli* has also a second enzyme that is able to excise 3mA—DNA-3-methyladenine glycosylase 1 (Tag), which is about 10-fold less active than AlkA [152]. In Gram-positive bacteria and single-celled eukaryotes, other glycosylases are also observed as a part of the BER pathway e.g., alkylpurine glycosylase D (AlkD), which excises bulky alkylpurine adducts and alkylpurine glycosylase C (AlkC), which deals mostly with 3mA [170,171].

The Fpg/Nei family of human glycosylases recognizes oxidized pyrimidines such as G or FapyA, hydroxyuracil (HydU), TG, and hydantoin lesions. In bacteria, Fpg and Nei scan the bacterial genome for any distortions of the DNA helix, especially oxidized pyrimidines [152]. While Fpg has no mammalian homologs, Nei corresponds with NEIL glycosylases. NEILs are bifunctional glycosylases that excise damaged base through β,δ-elimination. They are responsible for the excision of oxidative lesions such as 5hU, 5hC, and Fapy (8-oxo-G is not a preferred substrate). Two out of three NEIL isoforms identified in eukaryotes are present in mitochondria—NEIL1 (with 89 amino acid MTS), which repairs dsDNA, and NEIL2 (MTS length is not yet known), which repairs ssDNA [161]. NEIL glycosylases need a subsequent action of PNKP that removes 3′-phosphate and allows Polγ to work [155]. PNKP seems not to play a role in bacterial BER; however, studies show it is essential for RNA repair in many bacteria species [172].

Endonucleases are another group of BER enzymes. They are translocated into mitochondria in response to oxidative stress signals [173,174]. Mitochondrial APE1 (MTS not identified) and APE2 (15 amino acid MTS) are both homologs to exonuclease III (ExoIII) in *E. coli* [175]. ExoIII (also known as XthA) and endonuclease IV (EndoIV, also known as Nfo) excises ribose and allows PolI action during BER in bacteria. If any errors appear, the post-replication recombination is another scenario that bacteria use to repair their DNA.

Polymerases are crucial for the BER mechanism. In mitochondria, Polγ is commonly accepted as the only polymerase and well described [154]. The catalytic subunit of the enzyme has 3′—>5′ polymerase activity and 5′-deoxyribose lyase activity, which is essential for proper base excision and strand ends processing [176]. Polγ is yet another BER component similar to bacterial ones. It is a homolog of family A polymerases such as PolI from *E. coli* or polymerase from bacteriophage T7. Recently, other types of polymerases are reported in mitochondria [177,178,179,180]. An interesting example of novel polymerase is PrimPol [181,182]. It is responsible for replication maintenance. When bulky damage appears and replication is stalled, PrimPol can bypass those lesions (e.g., 8-oxo-G or AP sites) and restart replication. Cell lines lacking PrimPol show a slower mtDNA replication rate [181]. Interestingly, it belongs to the archaeal asparaginyl endopeptidase (AEP) superfamily of polymerases. Studies describe in human mitochondria also other types of polymerases (e.g., polymerase β), but results are inconclusive and still need further verifying [177].

The final element of the BER system is ligase. Bacterial and mitochondrial ligases belong to different subfamilies—mitochondrial LigIII is ATP-dependent, while LigA from *E. coli* needs a NAD^+^ as its cofactor. LigIII is essential for proper mtDNA repair. It also seems to play a role in mitophagy [183]. While in nDNA it acts in coordination with X-ray repair cross-complementing protein 1 (XRCC1), in mitochondria, this cooperation is not observed [184]. However, some authors describe XRCC1 present in mitochondria as a scaffold for other BER proteins (e.g., NTH1 or NEIL1) [183]. An additional protein related to BER is poly(ADP-ribose) polymerase 1 (PARP1). It forms a complex with LigIII and mtDNA and interacts with EXOG1, TFAM, and Polγ, serving as a negative regulator of repair processes [155].

Some human mitochondrial BER proteins do not have direct bacterial homologs described. However, other enzymes in bacteria may play those roles. For example, FEN1 endonuclease activity was first described in studies on bacterial polymerase complex, and now we know that bacteria use exonuclease activity of PolI instead of FEN1 during LP-BER [185]. In addition, PARP1 has no homologs in bacteria nor archaea—it may have been lost as a result of EGT during evolution [186]. On the other hand, some repair enzymes are highly conserved in many organisms. Human and bacterial UDG structures show identity in about 70% of amino acid residues [187]. Interestingly, enzymes of the BER system are also reported in archaea with high homology to eukaryotes. It may be considered as another piece of evidence of the archaeal origin of the ancestral host [188].

## 6. Conclusions

Mitochondria are crucial for maintaining the health of the cell and the integrity of the whole organism. Therefore, the origin of mitochondria and their similarity to bacteria have been discussed for many years. The theory of endosymbiosis is now well confirmed. It states that mitochondria originate from ancient α-proteobacteria that have been engulfed by the host’s cells over the course of evolution. Molecular machinery and mtDNA maintenance differ from their nuclear equivalents. It may yet be another argument in favor of their dissimilar origin. Similarities between human mitochondria and bacteria are observed in many aspects, ranging from the structure and physiology of the organelle to the construction and regulation of the genetic material. The BER system is a particularly interesting aspect to compare. As mitochondria are the site of OXPHOS, their genetic material is susceptible to oxidative DNA damage resulting from ROS action. Radicals may affect nucleobases in mtDNA and lead to subsequent mutations. Therefore, it is of crucial importance for the entire cell that the mitochondrion has effective repair systems (e.g., BER) which protect the integrity of mitochondrial genetic information. The majority of mtDNA mutations lead to degeneration diseases, e.g., in the heart and muscles. However, given the role of mtDNA in ATP production, mtDNA mutations may influence many other cell functions. As a result of its involvement in the most basic mitochondrial functionalities, mtDNA provides a lot of information about stress conditions affecting the cells. Moreover, taking into consideration that mitochondria are responsible for cellular respiration, any damage to mtDNA or other mitochondrial elements may cause long-term multisystemic failures and therefore lead to high morbidity and mortality [189]. To date, the main epidemiological factors of mitochondrial diseases remain still to be investigated and described. Mitochondria may contain both mutated and not mutated DNA molecules (heteroplasmy), which has far-reaching influence on cellular functions [78]. Specific symptoms of the disease and their intensity are highly dependent on the ratio of damaged to undamaged mtDNA molecules inside each mitochondrion. Moreover, the threshold of mtDNA heteroplasmy varies depending on the type of mutation and the type of tissue that is involved in the pathological process [78]. As a result, the patient may have some tissues with healthy and some tissues with mutated mtDNA. It causes variable symptoms throughout the course of the disease, which makes diagnosis more difficult [57]. Several hereditary diseases are mainly diagnosed based on family history due to a lack of diagnostic techniques that are able to cope with so many variables in the context of mtDNA mutations. Novel techniques are emerging, such as next-generation sequencing (NGS) or CRISPR/Cas9, which are promising in the context of mitochondrial disease studies [190]. Mitochondrial diseases are often associated with ATP synthesis defects; therefore, common symptoms include muscle weakness, cognitive defects, and brain degeneration, as all those cell types need a high amount of energy for proper functioning. It is roughly estimated that every 1 in 5000 people has some kind of mitochondrial mutation [10]. One of the most interesting is chloramphenicol toxicity, which results from mtDNA mutations. It is believed that mitochondrial sensitivity to antibiotics is related to its bacterial origin [191]. Examples of diseases related to dysfunctions in mitochondrial repair mechanisms and biochemical processes are presented in Table 2. 

Recently, mitochondrial disorders are also observed in relation to cancer development, neurodegenerative diseases, aging, and metabolic syndrome [78,197,198]. In the case of cancer, it is believed that mutations in mtDNA that lead to impaired respiration contribute to the propagation of cancerous phenotype. Moreover, the number of mtDNA copies have been correlated with different types of cancer. The decrease is reported for breast, liver, lung, and kidney cancers, while in prostate and head and neck cancers, excessive mtDNA copies are observed [199,200,201]. At the same time, cancer cell’s mitochondria are considered as therapeutic targets, which may be a feature of personalized medicine in the future [202]. As for neurodegenerative disorders, our understanding of Alzheimer’s and Parkinson’s disease is far from ideal. Up-to-date studies linked a higher level of DNA damage (e.g., resulting from inactive OGG1) with Alzheimer’s disease and mutations in genes coding for subunits of complex I of ETC with Parkinson’s disease [192,193,203]. Quite recently, “free radical theory of aging” was the most commonly accepted. It states that aging is related to mitochondria and toxic ROS action, which in time impair cellular integrity, leading to aging and subsequent cell death. The theory has been challenged over the years, and interestingly, in recent studies, aging was shown from the new perspective as the remnant of the symbiotic evolution of mitochondria [204]. The authors claim that apoptosis (resulting in excessive fission) undergoes a process resembling phagosomal lysis in *Rickettsia*. Moreover, novel observations correlate metabolic disease with mtDNA integrity—if repair enzymes of the BER system are inactive, it may increase the risk of obesity and insulin resistance [78]. Mitochondrial functions have also been connected with physiological stress where interdependence is discussed [205].

The origin of mitochondria is important for the full understanding of the pathologies that emerge with mtDNA mutations. The background of mitochondrial diseases is highly complex—the diagnosis is tricky due to many variable factors, and the experimental studies are challenging. Therefore, studies on mtDNA damage and mutation may benefit from contrasting perspectives. Having this in mind, we show that modern bacteria species are not as different from human mitochondria as previously thought. Common features at the molecular level should be taken into account, because choosing bacteria as the experimental model may be of great use in the field of mitochondrial disease studies. Mitochondrial repair mechanisms are the central “protector” of mtDNA integrity from oxidative DNA damage. Therefore, the full understanding of the mitochondrial BER system may contribute greatly to the advancement of mitochondrial disease therapies [10,30].

## Figures and Tables

**Table 1:** The BER system’s main proteins in human mitochondria and bacteria [151,155,161,162].

	*H. sapiens*	*E. coli*
DNA glycosylases		
UDG family	UNG1	Ung
		Mug
HtH family	NTH1	Nth
	OGG1	
	MUTYH	MutY
	MTH1	MutT
		MpgII
AAG family	AAG-A, AAG-B	AlkA
Fpg/Nei family	NEIL1, NEIL2	Fpg (mutM), Nei
AP endonucleases		
Xth family	APE1, APE2	ExoIII (XthA)
Nfo family		Endo IV (Nfo)
DNA polymerases		
Family A	Polγ	PolI
Family X	Polβ	
Family AEP	PrimPol	
DNA ligases		
ATP-dependent	LigIIIα	
NAD^+^-dependent		LigA
Other		
Flap endonuclease	FEN1	

**Table 2:** Examples of diseases related to mitochondrial repair system; FEN1—flap endonuclease, AMP—adenosine monophosphate, dRP—deoxyribose phosphate lyase, PNKP—polynucleotide kinase 3′-phosphatase, APTX—aprataxin, TDP1—tyrosyl-DNA phosphodiesterase 1, ATM—ataxia telangiectasia mutated, LigIII—ligase III, OMIM database—Online Mendelian Inheritance in Man [192,193,194,195,196].

Disease	Clinical Symptoms of Disease	Impaired Protein	Protein Function in Mitochondria
Huntington’s disease (HD) OMIM #143100	decrease in cognitive and motor functions	FEN1	Endonuclease, takes part in LP-BER
Microcephaly, seizures, and developmental delay (MCSZ) OMIM #613402	early infantile epileptic encephalopathy, cerebellar atrophy, peripheral neuropathy	PNKP	Occurs with Polγ and NEIL2, major 3′-phosphatase
Ataxia-oculomotor apraxia 1 (AOA1) OMIM #208920	cerebellar ataxia with peripheral axonal neuropathy, oculomotor apraxia, hypoalbuminemia	APTX	Removes 5′-AMP and 5′-AMP-dRP from DNA
Spinocerebellar ataxia with axonal neuropathy-1 (SCAN1) OMIM #607250	cerebellar atrophy, peripheral neuropathy, gait disturbance, sensory impairment	TDP1	Takes part in the repair of 3′-abasic sites and topoisomerase I-linked DNA adducts
Ataxia-telangiectasia (A-T) OMIM #208900	cerebellar ataxia, immune defects, cells are highly sensitive to ionizing radiation	ATM	Regulates mtDNA copy number, LigIII, and mitophagy

## Abbreviations

mtDNA	Mitochondrial DNA
nDNA	Nuclear DNA
OXPHOS	Oxidative phosphorylation
ROS	Reactive oxygen species
BER	Base excision repair
SP-BER	Short-patch BER
LP-BER	Long-patch BER
ETC	Electron transport chain
IMM	Inner mitochondrial membrane
OMM	Outer mitochondrial membrane
CL	Cardiolipin
PG	Peptidoglycan
D-loop	Displacement loop
Polγ	DNA polymerase γ
PolI	DNA polymerase I
POLRMT	DNA-dependent RNA polymerase
TFAM	Mitochondrial transcription factor A
LigIII	DNA ligase III
8-oxo-G	8-oxoguanine
SSB	Single-strand break
DSB	Double strand break
AP site	Apurinic/apyrimidinic site
SOD	Superoxide dismutase
CAT	Catalase
Gpx	Glutathione peroxidase
Trx	Thioredoxin
Prdx	Peroxiredoxin
GSH	Glutathione
OGG1	8-oxoguanine glycosylase
NEIL	Nei-like endonuclease
NTH1	Endonuclease III homolog 1
MUTYH	Human adenine DNA glycosylase
UDG	Uracil DNA glycosylase
UNG	Uracil N-glycosylase
APE1	AP endonuclease
PNKP	Polynucleotide kinase 3′-phosphatase
FEN1	Flap endonuclease
MTS	Mitochondrial targeting sequence
Fpg	Formamidopyrimidine-DNA glycosylase
Nei	Endonuclease VIII
AAG	N-methylpurine DNA glycosylase
AlkA	Alkyladenine DNA glycosylase
MutY	Adenine DNA glycosylase
MutT	8-oxo-dGTP diphosphatase
